# Supplementary material for: Association of Insomnia with 30-Day Postpartum Readmission: A Retrospective Analysis
Source: Int J Environ Res Public Health. 2023 May 25;20(11):5955. doi: 10.3390/ijerph20115955 (PMC10252679; doi:10.3390/ijerph20115955)
Supplement: Supplementary file 1 [file ijerph-20-05955-s001.zip › ijerph-2379115-supplementary.pdf]

Table S1: Distribution of Sociodemographic, Clinical, and Hospital Characteristics Among Index[a] Hospitalizations With and Without a Coded Diagnosis of Insomnia, Sensitivity Analysis Excluding Blood Transfusion From Definition of Maternal Comorbidities, Nationwide Readmissions Database, 2010-2019

Characteristic	No insomnia		Insomnia	
	**n[b]**	**%[c]**	**n[b]**	**%[c]**
**All Discharges**	34283319	100.0	26099	100.0
**Age (years)**				
15-19	2332004	7.0	979	4.0
20-24	7361734	22.1	3901	15.8
25-29	9851165	29.6	6864	27.8
30-34	9215203	27.7	8118	32.8
35-39	4488095	13.5	4859	19.7
40-49	1035117	3.0	1377	5.3
**Household income[d]**				
Highest quartile	7334019	21.6	6160	23.8
Third quartile	8529700	25.1	6876	26.6
Second quartile	8602286	25.3	6362	24.6
Lowest quartile	9511438	28.0	6454	25.0
**Primary payer[e]**				
Private	17892600	52.3	13911	53.4
Government	14720829	43.0	10977	42.1
Other	1601520	4.7	1181	4.5
**Elective admission**				
Non-elective	17229921	50.3	13610	52.2
Elective	17010816	49.7	12458	47.8
**Timing of admission**				
Weekday	27644201	80.6	21182	81.2
Weekend	6639118	19.4	4917	18.8
**Severity of illness[f]**				
Minor loss of function	21049316	61.4	7257	27.8
Moderate loss of function	10981063	32.0	13272	50.9
Major loss of function	2187426	6.4	5286	20.3

Extreme loss of function	62018	0.2	279	1.1
**Hospital location/type**				
Urban teaching	20747627	60.5	18293	70.1
Urban non-teaching	10050613	29.3	5612	21.5
Rural	3485078	10.2	2194	8.4
**Hospital bed size**				
Small	4751918	13.9	4238	16.2
Medium	9409904	27.4	6404	24.5
Large	20121497	58.7	15457	59.2
**Severe maternal morbidity[g]**				
No	34042512	99.3	25426	97.4
Yes	240807	0.7	673	2.6
**Obstetric comorbidity score[h], Mean (SD)**	4.9 (8.6)		16.0 (15.2)	

Abbreviation: SD, Standard Deviation

[a]Index events include delivery hospitalizations among patients aged 15-49 years who were at risk for 30-day readmission.

[b]Weighted to estimate national frequencies. The sum of subgroups may not add up to the total due to missing data.

[c]Column percentages may be used to compare the distribution of study characteristics in patients with and without insomnia.

[d]Determined by median household income for patient's zip code.

[e]Government includes Medicare and Medicaid. Other includes self-pay, no charge, and other payers.

[f]All patient refined diagnosis related group illness severity is estimated using a proprietary algorithm incorporating patient sociodemographics, comorbidities, and services rendered.[25]

[g]Severe maternal morbidity includes conditions captured by the Centers for Disease Control and Prevention[23] and excludes cases where blood transfusion is the only such condition.

[h]Non-transfusion obstetric comorbidity scores were calculated using a scoring system developed to predict severe maternal morbidity from hospital discharge data.[24]

Table S2: Sociodemographic, Clinical, and Hospital Characteristics Associated With 30-Day All-Cause Readmission Among Index[a] Hospitalizations, Sensitivity Analysis Excluding Blood Transfusion From Definition of Maternal Comorbidities, Nationwide Readmissions Database, 2010-2019

**Characteristic**	**Readmission Rate[b], % (95% CI)**	**Odds Ratio (95% CI)[c]**		
		**Crude model**	**Adjusted[d] Model 1**	**Adjusted[e] Model 2**
**All Discharges**	1.36 (1.34-1.39)			
**Age (years)**				
15-19	1.44 (1.40-1.48)	1.09 (1.06-1.12)	1.00 (0.98-1.02)	1.05 (1.03-1.07)
20-24	1.32 (1.29-1.35)	0.95 (0.93-0.96)	0.99 (0.98-0.96)	1.00 (0.98-1.01)
25-29	1.25 (1.23-1.28)	Reference	Reference	Reference
30-34	1.29 (1.27-1.32)	0.98 (0.96-0.99)	1.09 (1.08-1.11)	1.07 (1.06-1.09)
35-39	1.61 (1.57-1.65)	1.22 (1.20-1.24)	1.33 (1.31-1.35)	1.25 (1.23-1.27)
40-49	2.11 (2.04-2.18)	1.61 (1.57-1.65)	1.66 (1.62-1.70)	1.52 (1.48-1.55)
**Household income[f]**				
Highest quartile	1.15 (1.10-1.19)	Reference	Reference	Reference
Third quartile	1.29 (1.26-1.32)	1.13 (1.11-1.15)	1.12 (1.10-1.14)	1.10 (1.09-1.12)
Second quartile	1.37 (1.34-1.41)	1.20 (1.18-1.23)	1.18 (1.15-1.20)	1.15 (1.13-1.17)
Lowest quartile	1.58 (1.54-1.63)	1.39 (1.36-1.42)	1.29 (1.26-1.31)	1.24 (1.22-1.27)
**Primary payer[g]**				
Private	1.18 (1.15-1.21)	Reference	Reference	Reference
Government	1.60 (1.57-1.63)	1.36 (1.34-1.38)	1.33 (1.31-1.35)	1.29 (1.27-1.31)
Other	1.21 (1.16-1.27)	1.03 (1.00-1.06)	1.05 (1.02-1.08)	1.04 (1.01-1.07)
**Elective admission**				
Non-elective	1.31 (1.05-1.58)	Reference	Reference	Reference
Elective	1.41 (1.37-1.45)	0.95 (0.94-0.97)	1.02 (1.01-1.04)	1.03 (1.01-1.04)
**Timing of admission**				
Weekday	1.39 (1.36-1.42)	Reference	Reference	Reference
Weekend	1.25 (1.23-1.28)	0.90 (0.89-0.91)	0.90 (0.89-0.91)	0.92 (0.91-0.93)
**Severity of illness[h]**				
Minor loss of function	1.01 (1.00-1.03)	Reference	Reference	Reference
Moderate loss of function	1.64 (1.60-1.67)	1.62 (1.60-1.64)	1.59 (1.57-1.61)	1.36 (1.35-1.38)
Major loss of function	3.17 (3.08-3.27)	3.20 (3.15-3.25)	2.99 (2.95-3.04)	1.78 (1.75-1.81)

Extreme loss of function	7.40 (7.02-7.78)	7.80 (7.42-8.19)	7.01 (6.67-7.37)	2.21 (2.08-2.35)
**Hospital location/type**				
Urban teaching	1.46 (1.42-1.50)	Reference	Reference	Reference
Urban non-teaching	1.21 (1.18-1.24)	0.83 (0.81-0.85)	0.91 (0.89-0.93)	0.94 (0.93-0.96)
Rural	1.22 (1.18-1.25)	0.83 (0.81-0.85)	0.86 (0.84-0.88)	0.89 (0.86-0.91)
**Hospital bed size**				
Small	1.23 (1.17-1.30)	Reference	Reference	Reference
Medium	1.31 (1.27-1.35)	1.07 (1.03-1.10)	1.05 (1.02-1.08)	1.05 (1.02-1.09)
Large	1.42 (1.38-1.46)	1.15 (1.11-1.19)	1.11 (1.07-1.14)	1.10 (1.07-1.13)
**Insomnia**				
No	1.36 (1.34-1.39)	Reference	Reference	Reference
Yes	3.03 (2.69-3.38)	2.26 (2.03-2.53)	1.64 (1.47-1.83)	1.30 (1.16-1.45)
**Severe maternal morbidity**[i]				
No	1.34 (1.31-1.36)	Reference	N/A	Reference
Yes	5.29 (5.10-5.48)	4.12 (4.00-4.25)	N/A	1.44 (1.39-1.50)
**Obstetric comorbidity score**[j] **(10 points)**		1.451 (1.446-1.456)	N/A	1.292 (1.286-1.298)

Abbreviation: CI, Confidence Interval; N/A, Not Applicable
[a]Index events include delivery hospitalizations among patients aged 15-49 years who were at risk for 30-day readmission.
[b]Readmission rates calculated as the percentage of index hospitalizations readmitted within 30 days. Frequencies were weighted to estimate national readmission rates.
[c]Odds ratios and 95% confidence intervals were generated using survey-weighted logistic regression.
[d]Adjusted model 1 adjusts for all covariates included in the table except for severe maternal morbidity and obstetric comorbidity score.
[e]Adjusted model 2 adjusts for all covariates included in the table.
[f]Determined by median household income for patient's zip code.
[g]Government includes Medicare and Medicaid. Other includes self-pay, no charge, and other payers.
[h]All patient refined diagnosis related group illness severity is estimated using a proprietary algorithm incorporating patient sociodemographics, comorbidities, and services rendered.[25]
[i]Severe maternal morbidity includes conditions captured by the Centers for Disease Control and Prevention[23] and excludes cases where blood transfusion is the only such condition.
[j]Non-transfusion obstetric comorbidity scores were calculated using a scoring system developed to predict severe maternal morbidity from hospital discharge data.[24] Odds ratios reflect a 10-point change in score.
